# Adverse drug events in cost-effectiveness analyses of interventions for diabetic conditions: a scoping review protocol

**DOI:** 10.11124/JBIES-21-00460

**Published:** 2022-07-19

**Authors:** Mari Pesonen, Eila Kankaanpää, Virpi Jylhä

**Affiliations:** 1 Department of Health and Social Management, University of Eastern Finland, Kuopio, Finland; 2 Finnish Centre for Evidence-Based Health Care: A JBI Centre of Excellence, Helsinki, Finland

**Keywords:** adverse drug events, cost-effectiveness analysis, diabetes, diabetic macular edema, diabetic retinopathy

## Abstract

The objective of this review is to provide an overview of adverse drug events in cost-effectiveness analyses related to the pharmacological treatments of diabetes and diabetes-related complications.

**Introduction:**

The inclusion of adverse drug events in cost-effectiveness analyses is recognized in health technology assessments guidelines, but in practice, this is inconsistent. This inconsistency may affect the reliability of the evaluation and, therefore, indicate that the information provided for decision-making in health care is misleading. Reviewing if and how adverse drug events are incorporated in cost-effectiveness analyses is necessary to address this gap.

**Inclusion criteria:**

Studies including participants who are receiving pharmacological interventions for diabetes, diabetic retinopathy, or diabetic macular edema will be considered for inclusion. We will include sources that focus on cost-effectiveness analyses using modeling framework, and are published in English between 2011 and the present. Other types of analyses and other types of conditions will be excluded.

**Methods:**

The information sources to be searched include MEDLINE, CINAHL, Scopus, Web of Science, the NHS Economic Evaluations Database, and the Health Technology Assessment Database. Studies in English will be considered for inclusion in the review. Potential sources will be assessed by 2 independent reviewers and imported into the JBI System for the Unified Management, Assessment and Review of Information. The results of the search and the study inclusion process will be reported in a Preferred Reporting Items for Systematic Reviews and Meta-Analyses flow diagram. A specific data extraction form will be used to extract and analyze the data. Results will be presented in tabular and graphic formats with a narrative summary, and will be discussed in the context of current literature and guidelines.

## Introduction

Health care resources worldwide are finite, and decisions regarding their allocation are inevitable, prompting the need for evidence to guide the decision-making. Economic evaluation regarding pharmaceutical treatments is meaningful for enabling effective resource allocation with minimal waste. A cost-effectiveness analysis is a common way of performing economic evaluation in health care, as it compares the costs and the effectiveness between comparators, technically estimating the additional price for one additional unit of effectiveness. Usually, a modeling framework is feasible for enabling such an evaluation, as the longer follow-up period is more effective at catching rare adverse events and side effects in the analysis. The guidelines for the methodology of cost-effectiveness analysis and modeling highlight, for example, the importance of including all the relevant evidence of the health care interventions, a comparison of appropriate treatment alternatives, and assessment of the uncertainty associated with analysis and modeling.^1^,^2^ “All the relevant evidence” refers to information related to the costs and the effectiveness of an intervention from a certain perspective. Generally, that perspective is either from a societal or health service view, and the measure for effectiveness is quality-adjusted life years, which considers both the length and the quality of life. Therefore, all the direct and indirect costs, and all the factors influencing health-related quality of life should be addressed in the analysis and modeling.^3^

A health care intervention may achieve benefits but also cause harm, which should be observable in the analysis. The harm usually indicates adverse events (AEs) or complications that are undesirable and unintended experiences associated with the health care intervention. AEs have a wide range of severity and may be caused by an error (preventable) or occur despite proper treatment (non-preventable). Adverse drug events (ADEs) are specifically related to AEs associated with the whole process of pharmaceutical therapies. Other similar terms for ADEs are adverse drug reactions (ADRs) and adverse effects, and these are always causally related to the drug itself and, therefore, unavoidable. Thus, these non-preventable ADRs are also one form of ADE. All these complications result in a variety of outcomes, notably worsening of an existing pathology, lack of expected health improvement, outbreak of a new pathology, or a noxious response due to medication taken.^4^–^6^

Clearly, the treatment of ADEs causes direct costs, and ADEs are also associated with prolonged hospitalizations leading to additional societal costs in terms of productivity loss and poorer population health.^7^ Therefore, ADEs are a relevant part of the treatment affecting both the costs and the effectiveness of an intervention that should be distinctly considered in the cost-effectiveness analyses.

The appraisal committee of the National Institute for Health and Clinical Excellence^8^ recommends evaluating “the impact of benefits and adverse outcomes associated with the technology as seen from the patient's perspective”^(p.64)^ that explicitly delineate the meaningful role of adverse events in the analysis. Peryer *et al.* 9 conclude that, by not assessing ADEs, the analysis will lack balance and may make the intervention appear more favorable than it should. In turn, the Finnish guidelines for health technology assessments (HTAs) disclose that cost-effectiveness analyses should include ADEs that significantly affect the costs and the effectiveness of the intervention.^10^ These and many other guidelines for economic evaluation recognize the significance of ADEs, both in the costs and effectiveness estimates in the analysis,^11^,^12^ but provide insufficient instructions for practical implementation and methodology for the incorporation of ADEs into the analysis. Thus, these guidelines merely settle for noting the importance of ADEs, without concrete guidance for practice. The incorporation of ADEs in the cost-effectiveness analyses is important as they could affect the reliability of the results: an intervention with many ADEs would appear more positive if ADEs were omitted. Consequently, the ambiguous HTA guidelines may lead to problems in economic evaluation in practice.

The practices for the incorporation of ADEs in cost-effectiveness analyses are seemingly unclear and not all evaluations consider them. After performing a preliminary search of *JBI Evidence Synthesis*, MEDLINE, PROSPERO, Cochrane Library, and CINAHL, only a few reviews concerning the incorporation of ADEs in economic evaluation or cost-effectiveness analysis were identified.^13^–^15^ For example, Craig *et al.* 13 examined the incorporation of ADEs in economic models by conducting a survey for HTA reports commissioned by the National Institute for Health Research (NIHR). They concluded that only 54% of models included ADEs and, of these, 79% incorporated them by using a cost parameter and 67% by using a clinical (also known as effectiveness) parameter. In turn, Heather *et al.* 15 systematically investigated the inclusion of ADEs in economic decision analytic models of anti-tumor necrosis factor and concluded that current economic models have been unable to routinely or systematically consider the direct costs or consequences of ADEs. In addition, inconsistent practices for incorporation of ADEs are observable in different cost-effectiveness models for age-related macular degeneration^16^ as well as other ocular diseases, such as diabetic retinopathy and diabetic macular edema. Diabetic retinopathy is treated with hypoglycemics as the treatment of diabetic macular edema includes intravitreal anti-VEGF and/or corticosteroid injections, which can cause ADEs such as cataracts, intraocular pressure rise, retinal detachment, and vitreous haemorrhage^17^,^19^ When viewing cost-effectiveness analyses for diabetic macular edema, some of the cost-effectiveness analyses include ADEs^18^,^19^ and some do not.^20^,^21^ Diabetes is a risk factor for many ocular diseases and, interestingly, many cost-effectiveness analyses for diabetes appear to consider ADEs in both their cost and effectiveness estimates, which indicates overall variability in incorporation of ADEs across cost-effectiveness analyses for different diseases.^22^–^24^ Since the research into diabetes is broader compared with ocular diseases (eg, see Appendix I), the practices for economic evaluations may be more advanced and, thus, also incorporate ADEs more effectively. The incorporation of ADEs in the cost-effectiveness analyses across different diseases is an important topic that lacks systematic research.

Accordingly, the purpose of this study is to systematically investigate various cost-effectiveness analyses and their incorporation of ADEs, and thereby inform the practices of economic evaluation. The aim is to examine whether ADEs have been included in the model-based cost-effectiveness analyses for pharmacological interventions in patients with diabetes, diabetic retinopathy, and diabetic macular edema. By pharmacological intervention, we refer to a treatment with drug therapies.

As the current research into the topic is scarce, a scoping review was chosen to map the available evidence and contribute to the health economic research. A scoping review is suitable for examining how research is conducted on a certain topic with the aim to inform future research.^25^ Thus, a scoping review is feasible for examining whether and how ADEs are included in cost-effectiveness analyses.

## Review questions

Are ADEs included in the model-based cost-effectiveness analyses conducted for pharmacological interventions in diabetes, diabetic retinopathy, and diabetic macular edema? If so, how are these ADEs incorporated in the analyses?

## Inclusion criteria

### Participants

Studies including patients receiving pharmacological intervention for types 1 and 2 diabetes, diabetic retinopathy, or diabetic macular edema will be considered for inclusion. All other conditions will be excluded. No specific age range for the participants will be applied, nor will there be any limitations on the included pharmacological treatments.

### Concept

The principal concept of interest in this review is ADEs, including ADRs, because non-preventable ADEs are also often termed ADRs.^26^ This review examines the inclusion of ADEs caused by pharmacological interventions in cost-effectiveness analyses. Therefore, ADEs are the outcomes of this study but also a component of the scoping review's concept. The concept will not limit the search, as the exclusion of ADEs from the cost-effectiveness analyses is also a valid result of this review. The concept of interest examines whether and how ADEs are incorporated in cost-effectiveness analyses. The “how” examines whether the ADEs are incorporated in cost estimates and/or quality-of-life estimates and the rationale if they are omitted. Additionally, this review will consider the practical execution of the incorporation (eg, different health states for ADEs in the model/direct inclusion in the expected costs and quality-adjusted life years, and sources of the costs, disutilities, and incidences of ADEs), the reasoning behind the inclusion of ADEs (eg, thresholds for the severity or incidence of ADEs), and the possible discussion on ADEs and their impact on the results of the analysis. More detailed description of the “how” is available in the draft data extraction instrument in Appendix II.

### Context

There are no geographical nor any cultural or racial restrictions for the review.

### Types of studies

This review will include cost-effectiveness analyses, including cost-utility analyses, using an economic modeling framework. Cost-effectiveness analyses will only be considered if both evaluated treatments are pharmacological interventions or one of the treatments is a pharmacological intervention and the other is a placebo or a non-pharmacological treatment, such as laser. If none of the evaluated treatments is a pharmacological intervention, the cost-effectiveness/utility analysis will be excluded from the review. Only cost-effectiveness analyses and cost-utility analyses will be included in the review, because they include a treatment's impact on health-related quality of life. It is necessary to understand impact of ADEs on health-related quality of life before examining how health and the impact of ADEs can be valued in monetary terms. Therefore, other types of analyses addressing effectiveness in monetary terms, such as cost-benefit analyses, will be excluded. The review will include studies irrespective of their country of origin. The information collected will include the type of analysis (cost-effectiveness or cost-utility analysis); the treatments in comparison; the use of modeling framework and the type of modeling used; time horizon; perspective of the analysis; discounting, including the applied discount rate; the included costs; the patient-reported outcome measure used; and the result of the analysis. More detailed description of the types of sources is available in the draft data extraction instrument in Appendix II.

To analyze the evidence concerning ADEs in economic evaluation, this scoping review will consider published journal articles on cost-effectiveness or cost-utility analyses based on a modeling framework, and the National Institute for Health and Care Excellence (NICE), European Network for Health Technology Assessments (EUnetHTA), and International HTA database (INAHTA) technology appraisals. Systematic reviews and scoping reviews will be “hand searched” to identify published cost-effectiveness or cost-utility analyses matching the inclusion criteria. Only published analyses will be included to ensure they are of good quality and peer reviewed. Expert opinions will be excluded because of their provision of irrelevant information from the point of view of the research questions of this scoping review.

## Methods

The proposed scoping review will be conducted in accordance with the JBI methodology for scoping reviews.^25^

### Search strategy

The search strategy will aim to locate only published studies. An initial limited search of MEDLINE (PubMed) was undertaken to identify articles on the topic. The text words contained in the titles and abstracts of relevant articles and the MeSH-terms related to the keywords were used to develop a full search strategy (see Appendix I). The search strategy, including all identified keywords and index terms, will be adapted for each included database and/or information source. The reference list of all included sources of evidence will be screened for additional studies.

Studies published in English will be included, because most of the cost-effectiveness and cost-utility analyses are published in English. Only studies published since January 1, 2011, will be included, as relatively new data is needed in order to develop an overview of the current practices in economic evaluation.

The electronic databases to be searched include MEDLINE (PubMed), CINAHL (EBSCO), Scopus, Web of Science, NHS Economic Evaluations Database, and Health Technology Assessment Database. Sources of gray literature to be searched include the National Institute for Health and Care Excellence, EUnetHTA, and INAHTA technology appraisals.

### Study selection

Following the search, all identified citations will be collated and uploaded into Covidence (Veritas Health Innovation, Melbourne, Australia) and duplicates will be removed. Following a pilot test, titles and abstracts will then be screened by 2 or more independent reviewers for assessment against the inclusion criteria for the review. Potentially relevant sources will be retrieved in full and assessed in detail against the inclusion criteria by 2 or more independent reviewers. Reasons for exclusion of sources of evidence at full text that do not meet the inclusion criteria will be recorded and reported in the scoping review. Any disagreements that arise between the reviewers at each stage of the selection process will be resolved through discussion, or with an additional reviewer(s). The results of the search and the study inclusion process will be reported in full in the final scoping review and presented in a Preferred Reporting Items for Systematic Reviews and Meta-Analyses (PRISMA) flow diagram.^27^

### Data extraction

Data will be extracted from papers included in the scoping review by 2 or more independent reviewers using a data extraction instrument recording the source of evidence details, characteristics, and results. The data extracted will include specific details about the participants, concept (incorporation of ADEs), types of sources (model-based cost-effectiveness and cost-utility analyses), study methods, and key findings relevant to the review questions. The draft data extraction tool will be piloted using a representative sample of the studies to be reviewed. This representative sample consists of the 3 to 5 articles identified in the initial limited search of MEDLINE (PubMed).

A draft data extraction form is provided in Appendix II. The draft data extraction tool will be modified and revised as necessary during the process of extracting data from each included evidence source. Modifications will be detailed in the scoping review. Any disagreements that arise between the reviewers will be resolved through discussion, or with an additional reviewer. If appropriate, authors of papers will be contacted to request missing or additional data, where required.

### Data analysis and presentation

The PRISMA flow diagram will detail the study inclusion process. Characteristics of included studies will be presented in tabular format, including the information described in the data extraction form. Appendix III presents a more detailed data analysis plan, which provides a framework for the analysis and the presentation of the data.

The results will be displayed in both tabular and graphic presentations. The tabular presentation will examine the results related to the review questions (if ADEs are incorporated and how they are incorporated). The tables will present the characteristics and the results of the analyses that did not incorporate ADEs and the results of the analyses that did incorporate ADEs separately. A narrative summary will accompany the tabulated results. Within the tables and the narrative summary, we will qualitatively examine whether the attributes of sources (eg, disease, time horizon, patient-reported outcome measure, publication year) seem to affect the inclusion or exclusion of ADEs in cost-effectiveness and cost-utility analyses. For the cost-effectiveness and cost-utility analyses that included ADEs, the choice of which ADEs, the sources of ADEs, and the method for the incorporation of ADEs will be closely explored. Additionally, we will examine the possible sensitivity analyses performed for the cost-effectiveness and cost-utility analyses in relation to both numerical and descriptive results of the analysis. We will also collect information, to be reflected on in the discussion part of the review, on how the importance of ADEs for the results and the method of handling the ADEs in the model was discussed (see Appendix III).

## Acknowledgments

This review will contribute towards a degree award for MP.

## Funding

MP has received funding from Evald ja Hilda Nissi Foundation. The funders have no role in the review process.

## Author contributions

MP and EK identified the research topic and formed the research questions; MP wrote the protocol and had responsibility for the final content of the protocol; EK and VJ edited the protocol and advised on inclusion criteria, methods, and the overall style of the protocol; MP, EK and VJ read and approved the final protocol.

## Appendix I: Search strategy

### MEDLINE (PubMed)

Search conducted: February 28, 2022

**Table:**

Group	Individual search terms		Number of results for search terms (PubMed)	Searches (group 1 AND group 2)	Number of results for searches (PubMed)
1	#1	“diabetes mellitus” [MeSH Terms]	469,934	#1 AND #7	1988
	#2	“diabetes^∗^” [Title/Abstract]	600,274	(#1 OR #2) AND #7	3165
	#3	“diabetic retinopathy” [MeSH Terms]	27,305	(#1 OR #2 OR #3) AND #7	3165
	#4	“diabetic retinopathy” [Title/Abstract]	26,657	(#1 OR #2 OR #3 OR #4) AND #7	3202
	#5	“diabetic macular oedema” [Title/Abstract]	787	(#1 OR #2 OR #3 OR #4 OR #5) AND #7	3204
	#6	“diabetic macular edema” [Title/Abstract]	4117	(#1 OR #2 OR #3 OR #4 OR #5 OR #6) AND #7	3215
2	#7	“cost-effectiveness” ¤ [Title/Abstract]	68,475	(#1 OR #2 OR #3 OR #4 OR #5 OR #6) AND (#7 OR #8)	3306
	#8	“cost-utility” ¤ [Title/Abstract]	5653	(#1 OR #2 OR #3 OR #4 OR #5 OR #6) AND (#7 OR #8 OR #9)	3495
	#9	“economic evaluation” [Title/Abstract]	11,683	(#1 OR #2 OR #3 OR #4 OR #5 OR #6) AND (#7 OR #8 OR #9 OR #11)	3583
	#10	“economic model” [Title/Abstract]	2433	(#1 OR #2 OR #3 OR #4 OR #5 OR #6) AND (#7 OR #8 OR #9 OR #11 OR #15)	3907
	#11	“economic model^∗^” [Title/Abstract]	4147		
	#12	“CEA” ¤¤	Not relevant results		
	#13	“CUA” ¤¤	Not relevant results		
	#14	“Markov” [Title/Abstract]	26,005		
	#15	“Markov^∗^” [Title/Abstract]	29,125		
FINAL SEARCH STRATEGY: (“diabetes mellitus” [MeSH Terms] OR “diabetes^∗^” [Title/Abstract] OR “diabetic retinopathy” [MeSH Terms] OR “diabetic retinopathy” [Title/Abstract] OR “diabetic macular oedema” [Title/Abstract] OR “diabetic macular edema” [Title/Abstract]) AND (“cost-effectiveness” [Title/Abstract] OR “cost-utility” [Title/Abstract] OR “economic evaluation” [Title/Abstract] OR "economic model^∗^” [Title/Abstract] OR “Markov^∗^” [Title/Abstract])					3907
Filters			Number of results (PubMed)		
Years: 2011–2022			2705		
Language: English			2659		

¤ number of results is the same with/without “-”. ¤¤ CEA = cost-effectiveness analysis, CUA = cost-utility analysis.

## Appendix II: Draft data extraction instrument

**Table:**

**Scoping review details**
Scoping review title:
Review objective/s
Review question/s
**Evidence source details and characteristics (in relation to the population and sources of the scoping review)**
Citation details (eg, author/s, date, title, journal, volume, issue, pages)
Country (free text)
Population (details, eg, age/sex, disease, and population size)
Types of sources:
Type of analysis (CEA or CUA)
Comparators (free text)
Modeling method
Time horizon of the analysis (months/years)
Perspective of the analysis (societal/health care [irrespective of the funder])
Discounting applied in the analysis (yes/no, rate used %)
Costs included in the analysis (drug costs, follow-up costs, etc.)
PROM used for measuring quality of life or effectiveness (disease specific/general, PROM instrument)
Result of the CEA or CUA (ICER and qualitative conclusion of the result)
**Details/results extracted from source of evidence (in relation to the concept of the scoping review)**
Concept:
Are ADEs reported in the study that provides the efficacy estimates for the CEA/CUA^∗^? (yes/no)
If no: ADEs incorporated to the analysis? If yes: the source of ADE if not the study that provides the efficacy estimates to the analysis
If yes:
Definition of ADEs:
ADEs incorporated into cost-effectiveness/cost-utility analysis (yes/no)
If no: Reasons for not including ADEs in the analysis (free text)
If yes:
Details of the incorporation of ADE (eg, thresholds for severity/incidence for included ADEs, source of the incidence [RCT, expert opinion, systematic review etc])
ADEs in cost estimates (yes/no, how [in expected costs or ADE health state, source of costs])
ADEs in quality-of-life estimates (yes/no, how [disutility or ADE health state, source of the estimate])
Discussion of the ADEs and results (does the study discuss the role of ADEs in the analysis? Are ADEs addressed in the sensitivity analyses; if so, what their impact seems to be in relation to the numeric and descriptive results of the analysis)

ADEs, adverse drug events; CEA, cost-effectiveness analysis; CUA, cost-utility analysis; ICER, incremental cost-effectiveness ratio; PROM, patient-reported outcome measures; RCT, randomized controlled trial.

## Appendix III: A flowchart for the data analysis and collection of insights for the discussion

ADEs, adverse drug events; CEA, cost-effectiveness analysis; CUA, cost-utility analysis; ICER, incremental cost-effectiveness ratio; RCT, randomized controlled trial

## References

[1] Evers S Goossens M de Vet H van Tulder M Ament A. Criteria list for assessment of methodological quality of economic evaluations: Consensus on Health Economic Criteria. Int J Technol Assess Health Care 2005;21(2):240–245. 15921065

[2] Briggs AH Claxton K Sculpher MJ. Decision modelling for health economic evaluation. Oxford: OUP Oxford; 2006.

[3] European Network for Health Technology Assessment. Endpoints used for relative effectiveness assessment: health-related quality of life and utility measures. EUnetHTA; 2015. Report No. WP7.

[4] Council of Europe. Glossary of terms related to patient and medication safety. Committee of Experts on Management of Safety and Quality in Health Care (SP-SQS) Expert Group on Safe Medication Practices. World Health Organization; 2005.

[5] Skelly CL Cassagnol M Munakomi S. Adverse events. Treasure Island, FL: StatPearls Publishing; 2021. 32644389

[6] US Food and Drug Administration. Guidance for industry and investigators: safety reporting requirements for INDs and BA/BE studies [internet]. Center for Drug Evaluation and Research and Center for Biologics Evaluation and Research; 2012 [cited 2021 Oct 1]. Available from: https://www.fda.gov/regulatory-information/search-fda-guidance-documents/safety-reporting-requirements-inds-and-babe-studies.

[7] Rafter A Hickey A Condell S Conroy R O’Connor P Vaughan P. Adverse events in healthcare: learning from mistakes. Q J Med 2015;108:273–277. 10.1093/qjmed/hcu145 25078411

[8] National Institute for Health and Care Excellence. Guide to the methods of technology appraisal 2013 [internet]. NICE; 2018 [cited 2021 Oct 1]. Available from: https://www.nice.org.uk/process/pmg9/resources/guide-to-the-methods-of-technology-appraisal-2013-pdf-2007975843781. 27905712

[9] Peryer G, Golder S, Junqueira D, Vohra S, Loke YK. Chapter 19: Adverse effects. In: Higgins JPT, Thomas J, Chandler J, Cumpston M, Li T, Page MJ, *et al*., editors. Cochrane Handbook for systematic reviews of interventions. Version 6.1 [internet]. Cochrane; 2020 [cited 2021 Oct 1]. Available from: https://training.cochrane.org/handbook/current/chapter-19.

[10] Finnish Medical Agency. [Fimea's recommendation for assessing the therapeutic and economic value of medicines. Fimea develops, evaluates and informs publication series] [internet]. Finnish Medical Agency; 2012 [cited 2021 Oct 1]. Available from: https://www.fimea.fi/documents/160140/765540/21124_HTA-suositus_menetelma_ja_raportointisuositukset.pdf. [Finnish]

[11] Drummond MF Sculpher MJ Claxton K Stoddart GL Torrance GW. Methods for the economic evaluation of health care programmes. 4th edn. Oxford: Oxford University Press; 2015.

[12] Penaloza Ramoz MC Barton P Jowett S Sutton AJ. A systematic review of research guidelines in decision-analytic modeling. Value Health 2015;18:512–529. 26091606 10.1016/j.jval.2014.12.014

[13] Craig D McDaid C Fonseca T Christian S Duffy S Woolacott N. Are adverse effects incorporated in economic models? A survey of current practice. Int J Technol Assess Health Care 2010;26(3):323–329. 20584362 10.1017/S0266462310000371

[14] Craig D McDaid C Fonseca T Stock C Duffy S Woolacott N. Are adverse effects incorporated in economic models? An initial review of current practice. Health Technol Assess 2009;13(62):1–71. 10.3310/hta13620 20018146

[15] Heather EM Payne K Harrison M Symmons DPM. Including adverse drug events in economic evaluations of anti-tumour necrosis factor-a drugs for adult rheumatoid arthritis: a systematic review of economic decision analytic models. PharmacoEconomics 2014;32(2):109–134. 24338344 10.1007/s40273-013-0120-z

[16] Schmier JK Hulme-Lowe CK. Cost-effectiveness models in age-related macular degeneration: issues and challenges. Pharmacoeconomics 2016;34(3):259–272. 26563248 10.1007/s40273-015-0347-y

[17] Schmidt-Erfurt U Garcia-Arumi J Bandello F Berg K Chakravarthy U Gerendas BC. Guidelines for the management of diabetic macular edema by the European Society of Retina Specialists (EURETINA). Ophthalmologica 2017;237:185–222. 28423385 10.1159/000458539

[18] Brown GC Brown MM Turpcu A Rajput Y. The cost-effectiveness of ranibizumab for the treatment of diabetic macular edema. Ophthalmology 2015;122:1416–1425. 25935787 10.1016/j.ophtha.2015.03.032

[19] Pochopien M Beiderbeck A McEwan P Zur R Toumi M Aballéa S. Cost-effectiveness of fluocinolone acetonide implant (ILUVIEN®) in UK patients with chronic diabetic macular oedema considered insufficiently responsive to available therapies. BMC Health Service Res 2019;9(22). 10.1186/s12913-018-3804-4 PMC6327492 30626376

[20] Haig J Barbeau M Ferreira A. Cost-effectiveness of ranibizumab in the treatment of visual impairment due to diabetic macular edema. J Med Econ 2016;19(7):663–671. 26882365 10.3111/13696998.2016.1154566

[21] Kourlaba G Relakis J Mahon R Kalogeropoulou M Pantelopoulou G Kousidou O. Cost-utility of ranibizumab versus aflibercept for treating Greek patients with visual impairment due to diabetic macular edema. Cost Eff Resour Alloc 2016;14(7.):10.1186/s12962-016-0056-1 PMC4831170 27081372

[22] Gorgojo-Martínez JJ Malkin SJP Martín V Hallén N Hunt B. Assessing the cost-effectiveness of a once-weekly GLP-1 analogue versus an SGLT-2 inhibitor in the Spanish setting: Once-weekly semaglutide versus empagliflozin. J Med Econ 2019;23(2):193–203. 31613199 10.1080/13696998.2019.1681436

[23] Sabale U Ekman M Granström O Bergenheim K McEwan P. Cost-effectiveness of dapagliflozin (Forxiga^®^) added to metformin compared with sulfonylurea added to metformin in type 2 diabetes in the Nordic countries. Primary Care Diabetes 2014;9(1):39–47. 24840612 10.1016/j.pcd.2014.04.007

[24] Vega-Hernandez G Wojcik R Schlueter M. Cost-effectiveness of liraglutide versus dapagliflozin for the treatment of patients with type 2 diabetes mellitus in the UK. Diabetes Ther 2017;8(3):513–530. 28349443 10.1007/s13300-017-0250-y PMC5446377

[25] Peters MDJ, Godfrey C, McInerney P, Munn Z, Tricco AC, Khalil H, *et al.* Chapter 11: Scoping Reviews. In: Aromataris E, Munn Z, editors. JBI Manual for Evidence Synthesis [internet]. Adelaide: JBI; 2020 [cited 2021 Sep 2]. Available from: https://synthesismanual.jbi.global.

[26] National Research Council. Preventing medication errors: quality chasm series. Washington, DC: The National Academies Press; 2007; 36–38.

[27] Page NJ McKenzie JE Bossuyt PM Boutron I Hoffmann TC Mulrow JD. The PRISMA 2020 statement: an updated guideline for reporting systematic reviews. BMJ 2021;372:n71. 33782057 10.1136/bmj.n71 PMC8005924

